# A Contemporary Issue of Micro-Foundation of CSR, Employee Pro-Environmental Behavior, and Environmental Performance toward Energy Saving, Carbon Emission Reduction, and Recycling

**DOI:** 10.3390/ijerph18105380

**Published:** 2021-05-18

**Authors:** Hua Yu, Muhammad Salman Shabbir, Naveed Ahmad, Antonio Ariza-Montes, Alejandro Vega-Muñoz, Heesup Han, Miklas Scholz, Muhammad Safdar Sial

**Affiliations:** 1 School of Economics and Management, China University of Geosciences, Wuhan 430074, China; yuhuafei2013@cug.edu.cn; 2 Department of Management, College of Commerce and Business Administration, Dhofar University, Salalah 211, Oman; salman.shabbir55@gmail.com; 3 Faculty of Management Studies, University of Central Punjab, Lahore 54000, Pakistan; naveeddgk2010@gmail.com; 4 Social Matters Research Group, Universidad Loyola Andalucía, C/Escritor Castilla Aguayo, 414004 Córdoba, Spain; ariza@uloyola.es; 5 Public Policy Observatory, Universidad Autónoma de Chile, Santiago 7500912, Chile; alejandro.vega@uautonoma.cl; 6 College of Hospitality and Tourism Management Sejong University, 98 Gunja-Dong, Gwanjin-Gu, Seoul 143-747, Korea; 7 Division of Water Resources Engineering, Department of Building and Environmental Technology, Faculty of Engineering, Lund University, P.O. Box 118, 221 00 Lund, Sweden; miklas.scholz@tvrl.lth.se; 8 Civil Engineering Research Group, School of Science, Engineering and Environment, The University of Salford, Newton Building, Salford M5 4WT, UK; 9 Department of Town Planning, Engineering Networks and Systems, South Ural State University (National Research University), 76, Lenin prospekt, Chelyabinsk 454080, Russia; 10 Department of Management Sciences, COMSATS University, Islamabad 44000, Pakistan; safdarsial@comsats.edu.pk

**Keywords:** environmental performance, pro-environmental behavior, environmental footprints, China, corporate social responsibility, SME

## Abstract

The contemporary literature has largely addressed corporate social responsibility (CSR) at the macro or institutional level, whereas its effect at the micro-level is largely ignored. In addition, contemporary researchers have also ignored the importance of employee pro-environmental behavior to reduce the environmental footprint of small and medium enterprises (SMEs). With this background, the present study attempts to decrease the environmental footprint of the SME sector of China by implementing CSR at the micro-level through the involvement of employees because employees spend a significant amount of their time at workplaces, and hence their environment-related behavior may significantly contribute to improve the natural environment. In this regard, here we examined the impact of the micro-foundation of CSR on SMEs’ environmental performance with mediating effect of employees’ pro-environmental behavior. The data were collected from the different organizations in China. Our sample constitutes a supervisor–subordinate dyad from which we collected 562 filled questionnaires (281 from each). We used the structural equation modeling technique using AMOS software for data analysis, the results show that CSR, directly and indirectly, through employee’s pro-environmental behavior affects the environmental performance of SMEs, and employee pro-environmental behavior partially mediates this relationship. The findings of the present study are helpful for policymakers of the SME sector of China to address widespread environmental issues caused by their business operations.

## 1. Introduction

The phenomenon of globalization has put a lot of pressure on businesses to compete globally and survive in a stiff business environment [1]. As a result, modern businesses around the globe need to look for new ways to survive and expand their operations. 

Likewise, businesses are under pressure from different stakeholders who want enterprises to fulfill their needs in a manner that is environmentally friendly. Such pressures have forced businesses to respond in a socially responsible manner to the diverse set of needs of different stakeholders [2]. Corporate social responsibility (CSR) is a business strategy that helps to manage and meet the different needs of stakeholders. There are two primary concerns about CSR: what CSR means and why firms deal with CSR. In general, CSR is regarded as a voluntary entrepreneurial response beyond legal and economic responsibility [3]. However, CSR, to date, does not have a universal definition and is understood and interpreted differently by different scholars and organizations [4]. Carroll [5] defines a four-dimensional CSR that includes economic, legal, ethical, and philanthropic, where the first set of responsibilities is an economic and legal responsibility, the ethical responsibility is also important for businesses. In addition, there are several issues in the CSR domain, namely the closure of plants [6], Labor relations [7], human rights [8], rules to conduct the businesses [9], public relations [10], and environmental issues [11]. It was found that the business response to social, ethical, and environmental issues includes a variety of factors. There are different theoretical lenses to support the philosophy of CSR, for example, institutional theory [12], agency theory [13], and stakeholder theory [14]. 

During the last two decades, China has gained substantial economic development because of its massive production capability. China has been recognized as a global manufacturer in recent years, placing China as one of the leading economies in the world. Presently, China is the second-largest economy in the world, contributing almost 17 percent to world GDP [15]. But, unfortunately, such substantial economic development is attained at the expense of the environment and society, a critical drawback of the developing Chinese economy. Presently, China is the largest CO_2_ emitter in the world and emits more than 10 billion metric tons of CO_2_ per annum [16]. Whereas developed nations such as the United States of America (USA) and European Union (EU) have remarkably raised their environmental standards, and they have been investing a lot of resources in order to protect the environment, the biggest problem lies within the developing countries in Asia and Africa where environmental standards are far behind as compared to the USA and EU [17]. To further aggravate the situation, it is notable that the majority of the world’s manufacturing takes place in these two developing regions, which paints a bleak picture of the future and calls for emergency measures.

Small and medium-sized enterprises (SMEs) are valued the world over for their contribution to economic growth. However, their environmental impact is also important. For example, in the European region, SMEs are estimated to account for 64% of the total industrial pollution [18]. SMEs play an important role in many economies, especially in developing countries. SMEs relate to different industrial sectors around the world and make a significant contribution to job creation and world economic development. They constitute 90% of the world’s businesses and more than 50% of the employment. On average, SMEs contribute up to 40% of gross domestic product (GDP) in developing economies. These numbers are even higher when informal SMEs are also involved [19]. 

Unfortunately, SMEs around the world have been regarded as the entities that significantly add (60–70%) in global pollution which is alarming [20]. During the last decade, there was an immense increase in the number of Chinese SMEs, as they account for 99.8% of total legal enterprises of China in 2018. Likewise, the SME sector provided employment to more than 233 million individuals in the same year [21], but this is only one side of the coin, as the other is alarming because the SME sector of China is the largest contributor to China’s total industrial pollution. Industrial practices around the globe contribute significantly to increase the pollution level of the planet. As a result, the concern for environmental protection is an important issue for responding to environmental challenges [22]. In recent years, there has been a significant increase in greenhouse gases and CO_2_ emissions. Concerns about economic growth, the environment, and social relations are not new, but quite recently the researchers have shown a greater concern for businesses to act in a socially responsible manner. In the same vein, the importance of social responsibility has increased the need to do business in a variety of ways, combining the problems of business public action in a balanced, social and economic way [23]. 

The literature has long established that CSR activities are widely acknowledged worldwide, and CSR boundaries are ever-evolving [24]. CSR philosophy is having a significant impact on businesses due to the growing concern for the environment. Conventionally, enterprises were centrally focused on profit-making, but now the trend has been changed, as enterprises have been pushed by different stakeholders to consider the environment and work in an eco-friendly manner [25]. For many years, researchers evaluated financial outcomes for an organization, using CSR initiatives [26,27,28,29,30], but little attention has been paid to the impact of CSR in the context of environmental performance [31,32]. 

Therefore, the major aim of the current study is to investigate the relationship of CSR at the micro-level and environmental performance in the SME sector of China. The study also proposes that employees’ pro-environmental behavior mediates this relationship. The current study will address two specific research questions which are given as under:

RQ1: What is the impact of CSR at the micro-level on SMEs’ environmental performance? 

RQ2: whether the relationship between CSR at the micro-level and environmental performance is mediated by the pro-environmental behavior of the employees?

The current study adds significantly to the existing literature. To start with, the majority of previous studies on CSR have addressed the impact of CSR activities on the economic performance of the organization [28,29,30], whereas the relationship of CSR practices with environmental management is largely ignored [31]. In this regard, the current study is an important addition to the existing literature of CSR as it acknowledges the importance of CSR to improve the environmental performance of an organization. Second, the extant literature fails to indicate that the growing problem of environmental degradation cannot be solved only through good governance. Instead, environmental problems can be solved through individual efforts by getting employees involved to contribute positively to the environment through their volunteer environment-related behavior, we pose this volunteer behavior to protect the environment as employee pro-environmental behavior. Our argument here is that environmental responsibility is a matter of concern for all and it needs to be acknowledged at both the individual level and at a collective level. The SME sector of China provides employment to multi-million individuals of the country and if this large number of employees can be made self-responsible towards the environment, then thinking for a better and sustainable future is logical. Third, the extant literature has long explored the relationship of CSR at the macro level, such as organizational or industry levels, but the existing literature has largely ignored the impact of such micro CSR activities as the role of employees on the environmental performance of an organization. Lastly, studies provide sufficient evidence to deal with environmental degradation through the adoption of new technologies [33] improving the production processes [34], and introducing eco-friendly product models [35], but recognizing the role of each employee in minimizing the environmental degradation did not receive due attention in the recent literature. 

In order to bridge these gaps, the present study is pioneering because it attempts to explain the importance of CSR at the micro level by focusing on employees. Likewise, we contribute to the existing literature on CSR in the context of the SME sector of a developing country like China, whereas the previous studies largely addressed CSR in the context of developed nations [36,37,38]. Another contribution of the present study is to consider employee pro-environmental behavior as a mediator between the relationship of CSR and environmental performance. Lastly, the contribution of the present study in the context of the SME sector of China is of utmost importance as this sector is highly labor-intensive, and we argue that employees spend a lot of time at workplaces, so investigating the mediating effect of employee pro-environmental behavior to improve the environmental performance of an organization is imperative. The remainder of this article is divided into four major sections. The coming section deals with the theoretical framework and related literature for hypothesis development. Then comes the methodology part in which we have discussed sample, data collection process, and instrument. The last two sections deal with result and discussion sections in which we have drawn statistical results and then discussed these results in the light of the previous researchers.

## 2. Theoretical Framework and Related Literature

The current survey seeks support from two theories to formulate hypotheses. First, the theory of norm reciprocity proposed by Gouldner [39], who argues that when individuals receive some benefit from others, they are expected to return these benefits in a positive manner. In this aspect, the CSR engagement of an organization is well-received by the employees of an organization and they take the CSR engagement of their organization as an activity that benefits the whole society. Thus, being a member of the society, the employees are expected to reciprocate their organization positively. Different scholars have also used this theory to explain individual behavior [40,41,42]. Second, the social learning theory [43], is also relevant to the theme of current research in a way that when employees observe the CSR engagement of their organization, they are expected to learn this socially responsible behavior on their part too. Thus they also practice such behavior while they perform their job tasks at the workplace, and hence they behave in a socially responsible manner.

Organizations need to contribute to society and the environment by maximizing sustainable environmental and social interactions through CSR [44]. Employees are one of the most important partners to support progressive policies of an organization, including health security, financial security, and workplace comfort [45]. If an organization participates in CSR activities including the environmental domain of CSR, it triggers positive environmental behavior in employees [46]. Tian and Robertson [47] suggest that if organizations behave cohesively and share their CSR practices with employees, it urges employees to develop a sense of responsibility towards society. As a result, employees gain knowledge and a better understanding of what is happening in the environment and society and how their organizations contribute to protect the natural environment [48]. The supportive work environment, which is evident by CSR activities, is positively associated with the employees’ willingness to build and implement environmental related behavior. In this regard, Leitão, et al. [49], contend that supportive environment in an organization urges the employees to be engaged in behaviors that can reduce the environmental footprint of an organization. Thus, the employees are motivated to think about the ways through which Eco-innovation can be introduced at workplace as an important strategic enabler to reduce the environmental dilapidation. Open innovation can occur in an organization at both micro-level and macro level. However, at micro-level it is characterized by unique individual experiences and absorptive capacity of an individual. We, argue here that CSR activities of an organization create a supportive environment at workplace [7] which is well received by the employees as a motivator for eco-innovation at workplace. In learning environments, employees strive to organize their values with the organizational societal values [32]. This builds an organizational environment that promotes employees to develop a positive, volunteer behavior towards the environment [50]. Tian and Robertson [47] argue that employees’ understanding of environmental management activities of their organization affects their level of involvement to demonstrate pro-environmental behavior. When employees see their organizations as supportive towards environmental up-gradation, they feel encouraged to volunteer environmental behaviors [51]. Employees are inclined to reciprocate with organizational social and environmental activities and are expected to distribute this benefit voluntarily and in a discretionary manner [52]. The extant literature has long established that CSR contributes to emotional, behavioral, and attitudinal workplace factors [53,54,55]. To conclude, employees who know their organizations are more socially responsive are more likely to demonstrate pro-environmental behavior. Hence, it is not without logic to propose the following:

**H1.**  *CSR at the micro-level is positively related to employees’ pro-environmental behavior.*

The issue of environmental dilapidation has received considerable attention from scholars and policymakers in recent times. Different research studies about the deterioration of the natural environment are converging to the point that man-made activities have created a serious threat to the survival of our planet [56,57,58]. In response, societies all around the globe are becoming increasingly aware of the negative effects of environmental degradation on human health and life on Earth. A sustainable environment, defined as “living within the sustainable surroundings of the biosphere” [59], should be one of society’s most important goals to ensure long-term sustainable human life on Earth. Two approaches for achieving a sustainable environment include reducing current environmental degradation and adaptation of such practices that aim to limit the environmental footprint of different industries. 

A broad range of individual behaviors that help to maintain the environment is called pro-environmental behaviors, which may be further defined as individual behaviors that contribute to a sustainable environment, for instance, limiting energy consumption, waste avoidance, recycling, printing double side of the paper, and the environmental activism [60]. This behavior can be publicized (for example, using mass transit options and participating in different activities for environmental causes) or private (for example, composting, not using home air conditioning, and consuming less electricity). The positive behavior towards environment protection that individuals experience as part of their own life is a self-fulfilling prophecy that results from their own volunteer efforts [61]. Although integrated social and organizational structures may also support or hamper pro-environmental behavior. Despite the important role of employees in achieving better environmental efficiencies, the impact of personal behavior on environmental improvement has been largely studied in household settings [62,63], whereas the environmental contribution that individuals make to their working lives can be even more important since their actions can have a direct impact on the overall environmental performance of organizations [60]. Robertson and Barling [64], affirm that the key motivation that leads to increased levels of sustainable environment comes from a cohesive workforce that responds to a precarious vision expressed by business leaders seeking the value of people sharing by improving the environmental up-gradation methods.

Employees are key to an organization for achieving environmental performance since attaining a sustainable environment is an urgent matter for organizations to achieve the economic objectives in close coordination with the natural environment [65]. The actions of individuals, especially employees in organizations, can contribute significantly to the achievement or non-achievement of a sustainable environment. Recommended solutions to a wide range of environmental threats include a wide range of environmental efforts, such as the development of new sustainable products, processes, models, and technologies to reduce environmental dilapidation [66,67]. There have been some improvements in sustainable technologies, but researchers agree that the rate of sustainable development is slower than necessary to prevent further damage [68,69]. Organizations also acknowledge that technological delays and solutions to environmental problems may not be able to effectively protect their economy now and in the future [70]. Environmental dilapidation, as a form of negative human behavior, has posed a life-threatening challenge to the planet Earth [71]. Therefore, the task of organizations is to think of ways to care for employees and encourage them to practice good work ethics. CSR is one of the ways in which employees influence organizations with volunteer roles, choices, and social responsibility (assuming that employees’ behaviors hang on their views on the organization’s involvement in various CSR activities).

Organizations do not consume natural resources, do not generate waste, and do not produce pollution. Such choices are made, worked on, and required by managers, employees, and customers. On the other hand, organizations are not involved in ways to promote sustainability as employees at the workplace do. Therefore, the protection and elimination of a degrading environment should include a change in the generally unsettled behavior of employees. Sustainable environment goals can be achieved through the efforts of employees at all levels of the organization [72]. A general body of knowledge argues that CSR as an organizational indicator may affect employee behavior towards the environment. For example, Śmiechowski and Lament [73], reported that employees who consider their company as social and environmentally responsible are likely to develop contentious environmental behaviors. Employees who value their volunteer propensity towards the environment have a strong desire to stay in a company that shows sensitivity to protect the natural environment and are likely to communicate positively for their organization. In a recent study, it has been found that CSR is directly related to employees’ efforts for the environment [74]. Organizations that put emphasis on achieving environmental sustainability through CSR initiatives, are certain to reap better competitive advantages than those organizations that exercise it for the monetary reasons or to comply with state laws [75]. Gligor-Cimpoieru, et al. [76] suggested that visible support from organization has a positive effect on employee behavior, which in turn induces overall organizational performance including environmental performance. In a study Ji and Jan [77] concluded that CSR actions can increase emotional and behavioral outcomes of employees. In general, it is believed that with a certain set of CSR activities, employees can improve their pro-environmental behavior which ultimately leads to enhance organizational environmental performance.

To sum, CSR activities are likely to develop pro-environmental behavior among employees, and in turn, employees contribute significantly to enhance the environmental performance of an organization. When employees see their organization as an entity that benefits the environment, they are more likely to be affiliated with that company. Ultimately, they will be self-motivated to engage in behaviors that are intended to support the organization’s effort to achieve sustainable performance. Put simply, we argue that because employees are likely to associate themselves with the organizations that practice CSR proactively, it is likely that employees in such organizations will volunteer their efforts to preserve the natural environment, which in turn, contributes to the overall increase in the environmental performance of the organization. Hence we propose:

**H2.**  *Pro-environmental behavior positively relates to environmental performance.*

**H3.**  *CSR positively relates to environmental performance.*

**H4.**  *Pro-environmental behavior mediates between CSR and environmental performance.*

## 3. Methodology

### 3.1. Sample, Data Collection, and Handling of Common Method Bias

Data for the present study were collected from the SME sector of China. In doing so, we targeted three cities of China including Wuhan, Yichang, and Xiangyang in Hubei province. Before starting the actual data collection phase, we first contacted the spokespersons of different SMEs in these cities in order to perform an initial screening about the engagement of the organization in CSR activities. After carefully assessing the CSR activities, we prepared a list of organizations that were involved in CSR practices. After that, we randomly selected some organizations and ask the concerned authorities to cooperate in the data collection process. Those organizations which showed their initial consent were listed again for the final data collection phase. In this regard, we randomly selected 19 SMEs from these cities, which included different sectors such as textile, chemicals, footwear, electronic equipment, rubber, and others (See Table 1 for details). After initial screening and with a finalized list of organizations, we asked the concerned person of the selected organizations to indicate the individuals (supervisor–subordinate) for data collection. Those who were identified by the concerned authorities were contacted by us, and we clarified the purpose of our research to remove any ambiguity. 

Next, we carefully dealt with the issue of common method bias. For this purpose, we were in line with the recommendation of Podsakoff, et al. [78] to collect the data from different respondents. We, therefore, developed a dyad of supervisors–subordinates from selected organizations. Next, we distributed questionnaires to subordinates containing the information for variables, CSR, and environmental performance. The data for pro-environmental behavior were collected from supervisors, it is logical and seems appropriate to collect the data for variable pro-environmental behavior from supervisors because in most SMEs the supervisors work in close coordination with subordinates, and hence they can easily observe the pro-environmental behavior of their subordinates. Initially, we distributed 800 surveys (400 for supervisors and 400 for subordinates) among the respondents of selected organizations, and finally, we received 562 filled matched questionnaires (i.e., 281 supervisor–subordinate dyads) which were useful for data analysis. Table 1 presents the detailed information regarding the demographic of the sample and the type of industries.

### 3.2. Measures 

We used existing scales for measuring the variables of the present study, hence the reliability and validity of each instrument are pre-established. In this regard, we used the scale of CSR from Tian and Robertson [47]. The scale constituted a total of seven items (a sample item: *“My company makes investment to create a better life for future generations”*). Likewise, the scale of pro-environmental behavior was adapted from Blok*, et al.* [79], and this scale was comprised of a total of four items (a sample item*: “I switch off the lights and fan when not in use”*). Lastly, we adapted the scale of environmental performance from Zhu, et al. [80]. They used a total of five items to measure environmental performance (a sample item: “*my company has reduced its carbon emission”*). All the items were rated on a five-point Likert scale.

## 4. Results and Analysis

We performed the data analysis part of the present study in different steps. To begin with, we, first of all, conducted confirmatory factor analysis in order to know whether data is fitted to our theoretical model. In this regard, initially, we addressed some model fitting issues in order to get better results ( for instance some error terms had to be correlated), and eventually, we received better model fit results confirming that our theoretical model is well fitted to the data (χ2/df = 2.86, CFI = 0.92, GFI = 0.90, IFI = 0.92 and RMSEA = 0.071). We reported these results in Table 2. Next, we report the results of correlation, convergent validity (AVE values), discriminant validity (square root of AVEs), maximum shared variance (MSV), average shared variance (ASV), and reliability results (α and CR values). The results revealed that the correlation is positive and significant for all variables, and the values of correlations are within moderate ranges, which is an indication that there is no potential threat of multi-collinearity in our data. Furthermore, we also assess the convergent validity of our instrument by observing average variance extracted (AVE) values for all variables. The rule of thumb in this regard is if AVE> 0.5, then it means that convergent validity of the scale is established. In our case (see Table 2), all values were well above the threshold level of 0.5, hence convergent validity of all scales is well established. We also checked the reliability of our instrument by observing both Cronbach alpha (α) and composite reliability (CR) values. In this connection, both values were above 0.7 and in the acceptable range, hence verifying that the reliability of our instrument is established. We can also observe the results of discriminant validity from Table 2. To do so, we obtained square root values of all three constructs (shown as bold in Table 2) and compared them with the values of correlation of other variables. The rule here is that if the value of the square root of AVE for a variable surpasses comparative correlation values, it means that the items of each scale are discriminating with other scales, and hence discriminant validity is established.

### Hypotheses Testing 

We used the structural equation modeling (SEM) technique for hypothesis testing, which is an advanced level technique for analyzing complex models. The SEM is particularly effective when the framework includes a mediator like the present research. Moreover, SEM is a second-generation co-variance-based data analysis technique, which most contemporary scholars prefer to analyze the data at an advanced level [81,82,83] because this technique equips the researchers to estimate different interrelations in a single go. Whereas this feature of handling multiple regression analysis simultaneously was non-existent in traditional regression analysis. We performed our analysis in two steps. In the first step, we tested for direct effects, and in the second step, we tested for indirect effects (see Table 3) of our model. The direct effect model produced good model fit indices as the values of *χ*^2^/*df* = 2.96, CFI = 0.90, GFI = 0.88, IFI = 0.91 and RMSEA = 0.077 are within acceptable ranges, meaning that our direct effect model is producing good results and providing evidence that our data fits the model. Next, we tested the model fit results for our indirect model, which is our hypothesized model. In this case the values of *χ*^2^/*df* = 2.61, CFI = 0.93, GFI = 0.91, IFI = 0.96, and RMSEA = 0.069 are even better in comparison to our direct effect model (two factor), hence we established that our indirect effect model (three-factor) produced more suitable results. This is in line with our theoretical model and providing us proof that the relationship of CSR with environmental performance is better explained when we introduce pro-environmental behavior as a mediator. 

**Figure 1:**
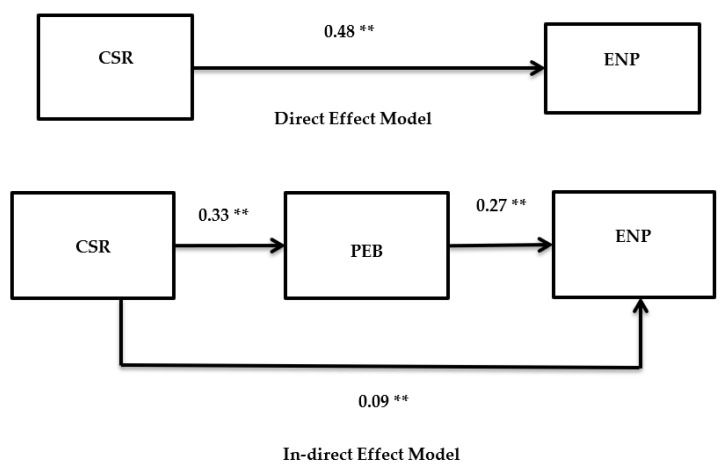
Direct effect model in upper pan, In-direct effect model in the lower pan. Note: ** = significant beta values (*p* < 0.05).

Next, we checked our results for hypotheses testing. For this purpose, we assessed beta values and resulting *p*-values to reach a decision. For example, we checked the effect of CSR on pro-environmental behavior (*β* = 0.33 ^**^, LLCI = 0.193, ULCI = 0.326, *p* < 0.05), pro-environmental behavior on environmental performance (*β* = 0.27, LLCI = 0.470, ULCI = 0.685, *p* <0.05) and CSR on environmental performance (*β* = 0.48, LLCI = 0.593, ULCI = 0.886, *p* <0.05). All these results provide sufficient grounds to accept our H1, H2, and H3. Finally, we tested mediation results by using bootstrapping in AMOS software. For this purpose, we used a large bootstrap sample of 2000, which produced significant results in favor of H4, and hence it is proved that our hypothesized model is statistically accepted (*β* = 0.090, LLCI = 0.338, ULCI = 0.510, *p* < 0.05). It is notable that for our H_4_ the beta value is reduced from 0.48 to 0.09, but still, it is significant, which means that pro-environmental behavior partially mediates between CSR and environmental performance (see Table 3). The authors have also reported these results of hypotheses in Figure 1. 

## 5. Discussion 

The results revealed that in terms of CSR efforts, employees play a crucial role, followed by other social and non-social interactions within organizations. In such cases, the value of the employees is also emphasized through the lens of stakeholder theory [84]. In order to implement a sustainable environment, an organization should focus more on its operations than its product, as already recommended by other scholars [85,86]. This was also confirmed by the philosophy of total quality management (TQM), which argues that if the process is improved, the products produced by an activity are sure to be the best [87]. In terms of performance, it appeared that if CSR is implemented carefully, it works as a booster to improve organizational performance especially the environmental dimension of organizational performance. Our study found that direct and indirect effects were significantly positive between CSR, PEB, and ENP.

Our results showed that the implementation of CSR programs results in better organizational performance due to the improved relationship between the organization and its partners, such as employees. In addition to confirming the positive correlation between CSR and environmental performance, the study also confirms an association between CSR and employee pro-environmental behavior. These findings are also supported by Nazari, Hrazdil, and Mahmoudian [32] and Kraus, Rehman, and García [31]. A review of the literature suggests that few studies have prodded on the impact of micro-foundation of CSR on individuals such as employees. Our study further affirms that there is a significant positive correlation between CSR and PEB, PEB, and ENP (mediating effect), indicating that when SMEs practice CSR activities, it motivates employees to be engaged in such behaviors that promote environmental sustainability. Given the positive effects of employee volunteer behavioral intentions to preserve the environment, it is imperative that this behavior should be promoted by SMEs. Although many studies have already begun to be identified for workplace environmental behavior, more attention should be paid to the actions and ways in which these factors encourage the environmental behavior of employees. Accordingly, we seek to facilitate extant literature by examining how CSR recognizes the impact of employees and their environmental behavior. The data from supervisor–subordinate dyads from the SME sector of China supported all hypotheses of the present study and confirmed our theoretical model. In particular, extending the micro-foundation of CSR in the settings of developing economies, our results indicate that when employees are aware of the moves of their organization towards society and nature, they are more likely to associate with their organization, which urges them to be involved in such behaviors that promote environmental sustainability, and one such behavior is pro-environmental behavior of employees.

The above argument can also be explained in the light of the theory of norm reciprocity in a way that the CSR engagement of an SME is regarded by the employees as an action that benefits society and the environment at large. Hence, as a member of society, the employees are motivated to reciprocate their organization positively and support their organization by involving themselves in discretionary behaviors. One such discretionary behavior is the pro-environmental behavior of employees at the workplace. Moreover, social learning theory also provides an explanation for why CSR activities of an SME urge the employees to behave in a socially responsible manner. In this vein, when employees observe the socially responsible behavior of their organization, they learn this behavior on their part too. Thus, in line with the theory of social learning, they become socially responsible while performing different tasks at the workplace.

### Limitations and Suggestions for Future

Our study is not without limitations, which in turn open new horizons for upcoming researchers in the same field. The first limitation of our study lies with the issue of a small sample and a concentrated geographic area. As the study only included 19 SMEs from six categories with a sample of 281 respondents, we think this sample and geographic concentration is insufficient to justify the generalizability of the results of the present study. To address this issue, future researchers are requested to go with a larger sample and involving more diverse geographic locations such as different cities of different provinces. Second, the cross-sectional nature of data limits the confidence of causality as it does not provide any historical information. Hence future researchers are required to develop a longitudinal survey strategy in order to develop better confidence in terms of causality. Third, our results are based on the perception of supervisor–subordinate dyads, so it will be better for future researchers to assess the actual performance of CSR initiatives on an organization by analyzing annual reports of the selected sample. Fourth,, there are some potential variables that need to be included in our proposed framework which could result in better results; we suggest including top management commitment towards the environment (as a moderator), eco-innovation (as a mediator), and co-worker advocacy towards environmental practices (as a moderator) as potential variables for future researchers. Lastly, the current study was carried out only at the micro-level and without controlling for different industries during analysis. Future researchers may conduct research that can blend both the micro-level and the macro-level, and control for different industry-related characteristics like organizational age, experience, etc.

## 6. Conclusions

Our study has some important implications for theory and practice. To begin with, it is important to note that relying on the majority with this hope that they will care for the environment is not sufficient to preserve nature. In this regard, SMEs are required to realize their important role in environmental management through the initiatives of CSR and involve their employees to practice eco-friendly behavior at the workplace. The average worker spends a lot of his daily time at his workplace. Therefore, the workplace becomes an important key for defining sustainable learning and development. In addition, workplaces can play a significant role in transferring pro-environmental behavior from the workplace to home life. In this regard, we argue that workplaces have the potential to translate environmental behaviors from work to daily life. These findings show that the workplace can serve as a useful forum for social change in the behavior of individuals for the environment, building effective collaboration between environmental educators and connecting with a strong, influential, and innovative community. In addition, SMEs have the opportunity to work with employees in close coordination to learn how they can contribute to preserving the environment.

As the economic concern is the primary focus of every organization, it is worth mentioning that pro-environmental behavior promotes employees feelings towards the environment and also serves as an economic enabler for organizations because when employees in the workplace consume less resources, it leads an organization towards better business efficiency, which ultimately affects business performance. Hence, it is to be noted by the policymakers of SMEs that developing a sense of eco-friendly behavior produces a dual output that works for environmental and economic cause for an organization. Likewise, CSR is a promising tool that promotes employee pro-environmental behavior at the workplace, as when employees see their organization is showing responsible behavior towards society and the environment, it promotes their volunteer intent to do the same at an individual level. Hence, this process collectively promotes organizational sustainable objectives.

Conversely, most Chinese SMEs are involved in CSR practices only to respond to state laws with this mistaken belief that investing in the environment is costly and is not wise to invest in an environmental cause beyond the legal obligations. This is the time to change this mistaken belief, as investing in environmental management asks for improving the overall efficiencies of a business which enhances the cumulative performance of an organization. Most SMEs in China also think that they belong to small-scale business and their contribution towards environmental degradation is negligible. Our research in this regard is an eye-opener because it sheds light to the fact the collective impact of the SME sector towards environmental degradation is alarming. We further argue that organizations especially in the SME sector of China need to arrange different seminars, workshops, and training sessions for their employees to increase their level of awareness regarding environmental issues and their contribution to slow down the process of environmental degradation. 

## Figures and Tables

**Table 1:** Demographic of the respondents (*n* = 281).

Demography	Frequency	Demography	Frequency
Age		Gender	
Supervisor		Supervisor	
28–33	72	Male	186
34–38	97	Female	95
39–43	68		
Above 43	44		
Subordinate		Subordinate	
22–27	84	Male	203
28–33	79	Female	78
34–38	66		
Above 38	52		
Experience		Type of industry	
Supervisor			
3–5	67	Rubber	41
6–10	146	Chemical	47
Above	68	Textile	53
Subordinate			
1–3	73	Footwear	44
4–6	152	Electronic equipment	63
Above	56	others	33

Notes: *n* = number of respondents.

**Table 2:** Correlations, validities, and reliabilities.

Variables	Mean	SD	CSR	PEB	ENP	α	CR	AVE	MSV	ASV
CSR	3.29	0.66	(0.79) ^b^	0.14 ^**^	0.26 ^**^	0.84	0.84	0.62	0.09	0.08
PEB	3.71	0.53		(0.83) ^b^	0.19 ^**^	0.79	0.81	0.67	0.14	0.12
ENP	3.84	0.68			(0.77) ^b^	0.87	0.88	0.59	0.10	0.07
(*χ*^2^/*df* = 2.86, CFI = 0.92, GFI = 0.90, IFI = 0.92 and RMSEA = 0.071) ^***^										

*** model fit indices for measurement model, n = 281, *p* < 0.001. CSR = corporate social responsibility, PEB = pro-environmental behavior, ENP = environmental performance, CR= composite reliability, AVE = average variance extracted MSV = maximum shared variance, ASV = average shared variance, ^b^ = square root of AVE, SD = standard deviation, ** = significant values (*p* < 0.05).

**Table 3:** Hypotheses testing.

Path	Beta Value	S.E	LLCI	ULCI	Decision
**Model 1: Standardized Direct effects**					
CSR→PEB (H_1_)	0.33 ^**^	0.123	0.193	0.326	Supported
PEB→ENP (H_2_)	0.27 ^**^	0.077	0.470	0.685	Supported
CSR→ENP (H_3_)	0.48 ^**^	0.156	0.593	0.886	Supported
(*χ*^2^/*df* = 2.96, CFI = 0.90, GFI = 0.88, IFI = 0.91 and RMSEA = 0.077) ^***^					
**Model 2: Standardized indirect effect (mediation model)**					
CSR→PEB→ENP (H_4_)	0.090 ^**^	0.031	0.338	0.510	Supported
(χ^2^ /df = 2.61, CFI = 0.93, GFI = 0.91, IFI = 0.96 and RMSEA = 0.069) ^***^					

CSR = corporate social responsibility, S.E= standard error, PEB = pro-environmental behavior, ENP = environmental performance, LLCI = lower limit confidence interval, ULCI = upper limit confidence interval, *** model fit indices for measurement model, ** = significant values (*p <* 0.05).

## Data Availability

The data will be made available on request from the corresponding author.
